# Biomarkers in QuantiFERON Supernatants May Help Distinguish Latent Tuberculosis Infection From Active Tuberculosis: Preliminary Report

**DOI:** 10.1155/mi/1686665

**Published:** 2026-07-15

**Authors:** Dagmara Borkowska-Tatar, Anna Zabost, Michał Czopowicz, Ewa Augustynowicz-Kopeć

**Affiliations:** ^1^ Department of Microbiology, National Reference Laboratory for Mycobacteria, Institute of Tuberculosis and Lung Diseases, Płocka Street 26, Warsaw, 01-138, Poland; ^2^ Division of Veterinary Epidemiology and Economics, Institute of Veterinary Medicine, Warsaw University of Life Sciences-SGGW, Nowoursynowska Street 159c, Warsaw, 02-776, Poland, sggw.pl

**Keywords:** host immune response, interferon gamma (IFN-γ), interleukin-7 (IL-7), tuberculosis diagnostics

## Abstract

**Background:**

Latent tuberculosis infection (LTBI) is an important public health problem and a major source of future active tuberculosis (ATB) cases. In this preliminary study, we sought to identify host biomarkers that could potential differentiate between LTBI and ATB.

**Methods:**

A total of 145 plasma samples obtained from antigen tubes of the QuantiFERON‐TB Gold Plus assay were analysed. Concentrations of 20 selected cytokines were determined using a multiplex bead‐based immunoassay (Luminex xMAP Technology). Cytokine profiles were compared between patients with ATB, LTBI, and non‐tuberculosis (TB)/LTBI controls. Diagnostic performance was assessed using receiver operating characteristic (ROC) analysis and multivariable logistic regression modelling.

**Results:**

Comparison of cytokine profiles showed significant differences in 13 of 20 analysed markers between LTBI and ATB. Among all evaluated biomarkers, interleukin‐7 (IL‐7) demonstrated the highest diagnostic potential in differentiating the two conditions (95.9%), outperforming interferon‐gamma (IFN‐γ), the key component of current IFN‐γ release assay (IGRA) assays. A logistic regression model based on IL‐7 and IFN‐γ achieved high diagnostic accuracy (98.1%) in this study cohort.

**Conclusion:**

These preliminary findings suggest that IL‐7, in combination with IFN‐γ, may serve as a promising candidate biomarker signature for distinguishing LTBI from ATB. Nevertheless, these findings are based on a single‐centre dataset and represent internal, non‐externally validated results. Therefore, external validation in independent multicentre cohorts is essential before any clinical application can be considered.

## 1. Introduction

According to the World Health Organization (WHO) estimates, latent tuberculosis infection (LTBI) affects approximately 25% of the world’s human population, equivalent to nearly 2 billion people [1, 2]. This represents a huge reservoir of potential future cases of active tuberculosis (ATB) and one of the key public health challenges globally [3].

The risk of progression of LTBI to ATB averages 5%–10% over a lifetime, with the highest risk observed in the first 2 years after infection [2, 3]. The risk is markedly elevated among immunocompromised individuals, especially those treated with immunosuppressive drugs (including TNF‐α inhibitors), organ transplant recipients, patients with haematologic malignancies, chronic kidney disease, diabetes, and among children younger than 5 years of age [3, 4].

The epidemiology of LTBI shows significant regional variation closely correlated with the incidence of ATB. The greatest prevalence of LTBI occurs in high‐burden countries, notably in sub‐Saharan Africa, Southeast Asia, and the Western Pacific region [1, 3]. In countries with low ATB incidence, such as in Western Europe, North America, and Australia, LTBI occurs less frequently but is concentrated in defined risk groups, including migrants from high‐endemic regions, prisoners, homeless populations, and medical personnel [1–4].

The global tuberculosis (TB) problem is further exacerbated by the increasing drug resistance of *Mycobacterium tuberculosis*, HIV co‐infection, and adverse socio‐economic and geopolitical conditions such as armed conflict, population migration, and limited access to health care [3, 5]. These factors not only increase the transmission of mycobacteria but also hinder the effective detection, monitoring, and treatment of both ATB and LTBI [3–5].

As people with LTBI are a major source of future ATB cases, an elimination strategy requires reliable identification and treatment of latent infection [2–4]. However, the diagnosis of LTBI remains a challenge as the infection is asymptomatic and the results of standard microbiological and imaging studies are negative. According to the WHO definition, LTBI is a state of sustained immune response to *M. tuberculosis* antigens in the absence of clinical, radiological and microbiological features of ATB [2, 4].

Effective identification and treatment of LTBI is now recognised as one of the pillars of the WHO’s global ‘End TB’ strategy, which aims to significantly reduce TB morbidity and mortality by 2035 [3, 6].

Despite the availability of various diagnostic methods, such as tuberculin skin test (TST), interferon gamma release assay (IGRA) and tuberculosis antigen‐based skin tests (TBST), a positive result from any of these does not clearly distinguish LTBI from ATB, nor can it predict when or if the active disease will develop [7]. Therefore, there is a need to identify new biomarkers of LTBI and ATB and to develop tests that can accurately differentiate between the two conditions and assess the risk of infection progression [8, 9]. In response to *M. tuberculosis* infection, the host organism secretes a number of pro‐inflammatory cytokines, both in the organs and in the peripheral blood, and their concentrations can be relatively easily determined [10, 11].

In this context, the aim of the present study was to identify host‐derived cytokine biomarkers with potential diagnostic value in differentiating LTBI from ATB using a multiplex immunoassay approach.

## 2. Materials and Methods

The study was conducted in accordance with the Declaration of Helsinki. The study protocol was approved by the Bioethics Committee at the National Institute of Tuberculosis and Lung Diseases in Warsaw (Approval Number KB‐10/2023). Before participating in the study, all participants provided written informed consent. All data were anonymized before the analysis.

### 2.1. Sample Collection and Data Retrieval

145 plasma samples obtained from TB1 and TB2 antigen tubes were analysed after QuantiFERON‐TB Gold Plus (QFT‐Plus) testing. Following standard incubation and centrifugation performed according to the manufacturer’s instructions, the resulting plasma was stored under recommended conditions until further analysis [12]. Following standard incubation and centrifugation performed according to the manufacturer’s instructions, the resulting plasma was stored under recommended conditions until further analysis [12]. The study cohort was retrospectively assembled from samples submitted for routine diagnostic testing at the National Reference Laboratory for Mycobacteria, National Tuberculosis and Lung Diseases Research Institute in Warsaw, between 2022 and 2025. No additional participant recruitment was performed, and all samples meeting the inclusion criteria, namely, the availability of residual plasma obtained after QFT‐Plus testing and the availability of microbiological and/or clinical data enabling assignment to the respective study group: ATB, LTBI or the control group, were included in the analysis during the study period.

1. ATB group (*n* = 37)—samples from patients with microbiologically confirmed TB. Both 27 QFT‐Plus‐positive patients and 10 QFT‐Plus‐negative patients were included in this group.
2. LTBI group (*n* = 57)—samples from QFT‐Plus‐positive individuals in the absence of microbiological and/or clinical confirmation of TB.
3. Control group (*n* = 51)—samples from individuals with a negative QFT‐Plus result and no available microbiological or clinical evidence suggestive of ATB or LTBI.

This classification enabled comparative assessment of cytokine profiles across distinct three groups of distinct immunological states associated with exposure to *M. tuberculosis* (Table 1). Given the retrospective nature of the study and the use of a routine diagnostic cohort, the possibility of selection bias cannot be excluded.

**Table 1:** Demographic characteristics and QuantiFERON‐TB Gold Plus results of 145 patients in three groups.

Characteristic	ATB group (*n* = 37)	LTBI group (*n* = 57)	Control group (*n* = 51)	*p*‐Value
Demographic characteristics				
Men^a^	32/37 (86.5%)	33/57 (57.9%)	23/51 (45.1%)	<0.001
Age (years)^b^	50, 31–64 (8–73)	42, 12–66 (1–89)	40, 29–56 (3–83)	0.280
Children (<18 year‐old)^a^	3/37 (8.1%)	24/57 (42.1%)	9/51 (17.7%)	0.003
QuantiFERON‐TB Gold Plus results				
TB1 (IU/mL)^b^	1.10, 0.17–3.54 (0–9.70)	0.79, 0.43–2.90 (0–9.91)	0.03, 0–0.23 (0–0.34)	—
TB2 (IU/mL)^b^	1.53, 0.24–3.62 (0–9.86)	0.80, 0.43–3.43 (0.02–9.91)	0.03, 0–0.21 (0–0.34)	—
TB_max_ (IU/mL)^b^	1.53, 0.31–3.82 (0–9.86)	0.87, 0.50–3.43 (0.35–9.91)	0.06, 0.01–0.27 (0–0.34)	—
QFT‐plus positive	27/37 (73.0%)	57/57 (100%)	0/51 (0%)	—

*Note:* TB1 = interferon gamma value in the QuantiFERON‐TB Gold Plus antigen tube containing peptides stimulating CD4^+^ T cells; TB2 = interferon gamma value in the QuantiFERON‐TB Gold Plus antigen tube containing peptides stimulating both CD4^+^ and CD8^+^ T cells; TB_max_ = the highest interferon gamma value (IU/mL) in the QuantiFERON‐TB Gold Plus antigen tubes TB1 or TB2.

Abbreviations: ATB, active tuberculosis; LTBI, latent tuberculosis infection; NON‐TB/LTBI, non‐tuberculosis/latent tuberculosis infection; QFT‐Plus, QuantiFERON‐TB Gold Plus test.

^a^ presented as count and proportion and compared between groups using maximum likelihood *G* test.

^b^presented as median, interquartile range (IQR), and range.

### 2.2. Bead‐Based Multiplex Assay

Cytokine concentrations were determined using the Bio‐Plex Pro Hu Immunotherapy Panel, 20‐Plex kit (Bio‐Rad, Hercules, USA) using Luminex xMAP technology, combining the properties of classical ELISA with flow fluorescence cytometry. The choice of the panel was dictated by the wide range of immunological markers described in the literature as potentially useful in differentiating between TB and LTBI. The panel included the following markers: granulocyte‐macrophage CSF (GM‐CSF), interferon (IFN)‐γ, interleukin (IL)‐2, IL‐4, IL‐5, IL‐6, IL‐7, IL‐8/CXCL8, IL‐10, IL‐13, IL‐15, IL‐17A, IL‐18, 10 kDa IFN‐γ‐induced protein (IP‐10/CXCL10), monocyte chemoattractant protein (MCP‐1/CCL2), monokine induced by IFN‐γ (MIG/CXCL9), macrophage inflammatory protein (MIP)‐1α/CCL3, MIP‐1β/CCL4, regulated on activation normal T‐cell expressed and secreted (RANTES/CCL5), tumour necrosis factor (TNF‐α). The assays were performed according to the manufacturer’s instructions [13].

The basis of the assay was fluorescent magnetic beads, each containing a unique combination of fluorescent dyes. Antibodies directed against one of the measured cytokines were immobilised on the surface of each of the 20 bead types; during incubation with plasma samples, the cytokines were specifically bound through the antibody–antigen affinity. After labelling with a fluorochrome (phycoerythrin), cytokines bound on the beads were quantitatively detected in an analyser using an advanced laser system. Results are presented as median fluorescence intensity (MFI) values, which were converted to cytokine concentrations [pg/mL] based on standard 5‐parameter logistic calibration curves [13]. Concentrations below the lower limit of quantification (LLOQ) and upper limit of quantification (ULOQ) were replaced by the direct estimates from the 5‐parameter logistic curve and by the ULOQ, respectively.

### 2.3. Statistical Methods

Numerical variables were examined for normal distribution using normal probability *Q*–*Q* plots and the Shapiro–Wilk *W* test. As the normality assumption was violated in all cases, numerical variables were summarised using the median, interquartile range (IQR), and range. Numerical variables were compared between unpaired groups using the Mann–Whitney *U* test and between paired groups with the Wilcoxon’s signed‐rank test. Correlations between numerical variables were determined using Spearman’s rank‐order correlation coefficient (*R*_s_). Categorical variables were presented as counts (*n*) and proportions (%) in groups. The 95% confidence intervals (CIs 95%) for proportions were calculated using the Wilson’s score method. Categorical variables were compared between unpaired groups using the maximum likelihood *G* test. The accuracy of cytokine concentrations and logistic models in the classification of patients to LTBI groups was assessed by computing the area under the receiver operating characteristic curve (AUROC). AUROC was interpreted as follows: >90%—an excellent test, 81%–90%—a good test, 71%–80%—a fair test, and ≤70%—a poor test [14]. The optimal cut‐off value was determined by maximisation of Youden’s index (*J*). At an optimal cut‐off value, the diagnostic sensitivity (Se), specificity (Sp) (referred to as relative Se and relative Sp if the imperfect test was used as a reference standard), as well as the positive and negative likelihood ratios (LR+ and LR−), were calculated. Due to multiple comparisons performed in the univariable analyses, the Holm–Bonferroni correction was applied to the significance level (α) of 0.05. The cytokines which proved significant in the univariable analysis and which had the highest accuracy were entered into the multivariable analysis. The multivariable analysis was performed using the multiple logistic regression run according to the backward stepwise procedure. Goodness‐of‐fit of the models was evaluated using the Hosmer–Lemeshow *χ* ^2^test (H–L *χ* ^2^) and Nagelkerke’s pseudo‐*R* ^2^ coefficient [15]. The effect size was expressed as the odds ratio (OR). Statistical analysis was performed in TIBCO Statistica 13.3 (TIBCO Software Inc., Palo Alto, CA, USA).

## 3. Results

### 3.1. Analysis of Cytokine Concentrations in TB1 and TB2 Tubes of the QuantiFERON‐TB Gold Plus

There were no significant differences in cytokine concentrations between tubes TB1 and TB2, and concentrations from both tubes were highly correlated (*R*_*s*_ = 0.71–0.96; Table 2). Therefore, the highest concentration of a given cytokine in tube TB1 or TB2 (TB_max_) was used in further analyses.

**Table 2:** Comparison of 20 cytokine concentrations between tube TB1 and TB2.

Cytokine (pg/mL)	LLOQ (pg/mL)	ULOQ (pg/mL)^a^	Number of samples <LLOQ	Number of samples >ULOQ	Tube TB1	Tube TB2	Wilcoxon’s signed rank test *p*‐value^b^	*R*_s_^c^
GM‐CSF	0.48	7846	–	–	19.2, 11.2–36.2 (2.42–58)	18.2, 11.9–36.9 (3.74–73.1)	0.792	0.934
IFN‐γ	1.57	25,665	–	–	297, 158–416 (28.3–1141)	273, 169–391 (35–1359)	0.647	0.929
IL‐2	1.29	21,178	–	–	123, 73.8–206 (20.7–1387)	125, 71.7–201 (20.3–1328)	0.786	0.936
IL‐4	0.19	3064	–	–	26.3, 19.7–55.6 (2.19–177)	25.1, 19.6–58.5 (7.01–177)	0.344	0.947
IL‐5	3.63	59,499	–	–	370, 186–637 (19.1–1367)	343, 186–601 (28.6–1228)	0.878	0.915
IL‐6	0.38	6244	–	TB1: 1 TB2: 0	1223, 367–2171 (7.37–25000)	1043, 370–2009 (7.37–12155)	0.065	0.861
IL‐7	1.92	31,475	–	–	36.4, 13.8–63.5 (5.86–208)	36.4, 16.8–63.5 (1.45–203)	0.955	0.789
IL‐8/CXCL8	0.85	13,992	–	TB1: 42 TB2: 36	26,335, 12,491–56,000 (2271–56,000)	27,805, 13,434–56,000 (2460–56,000)	0.777	0.711
IL‐10	1.06	17,427	–	–	17.9, 11.3–29.9 (2.75–89.4)	17.7, 9.83–24.9 (2.51–101)	0.003^d^	0.871
IL‐13	0.31	5157	TB1: 39 TB2: 40	–	1.92, 1.10–3.51 (0.089–97.6)	1.92, 1.10–3.51 (0–71.7)	0.368	0.872
IL‐15	12.42	203,426	–	–	639, 507–1329 (224–2887)	653, 497–1292 (213–2899)	0.551	0.929
IL‐17	2.44	39,972	TB1: 1 TB2: 0	–	113, 80.2–319 (8.65–707)	115, 82.5–310 (37.9–760)	0.632	0.934
IL‐18	0.66	10,892	–	–	83.7, 52.4–113 (11.1–450)	79.4, 54.2–110 (16.3–353)	0.865	0.923
IP‐10/CXCL10	3.41	34,953	–	TB1: 2 TB2: 2	5691, 2581–12,079 (201–140,000)	6436, 2674–12,388 (394–140,000)	0.169	0.962
MCP‐1/CCL2/MCAF	0.53	8755	–	–	2893, 1527–4489 (74–22,106)	2618, 1438–4283 (67.2–20,179)	0.008^d^	0.876
MIG/CXCL9	3.16	32,365	–	TB1: 2 TB2: 2	5664, 2314–13,046 (336–130,000)	5397, 2398–14,923 (412–130,000)	0.029^d^	0.963
MIP‐1α/CCL3	0.12	1218	–	TB1: 2 TB2: 1	666, 301–1244 (9.53–4900)	614, 290–1089 (7.79–4900)	0.118	0.863
MIP‐1β/CCL4	1.41	1439	–	TB1: 5 TB2: 5	1921, 1103–2750 (462–5800)	1786, 1195–2595 (411–5800)	0.056	0.874
RANTES/CCL5	16.72	26,467	–	TB1: 2 TB2: 9	9792, 5969–16,673 (391–106,000)	8723, 5043–15,642 (322–106,000)	0.008^d^	0.919
TNF‐α	3.33	54,566	–	–	1318, 739–2401 (76.8–11,748)	1364, 714–2220 (129–15,263)	0.238	0.910

*Note:* Concentrations presented as median, interquartile range (IQR), and range. IL‐8/CXCL8, interleukin‐8; IP‐10/CXCL10, 10 kDa IFN‐γ‐induced protein; MCP‐1/CCL2, monocyte chemoattractant protein; MIG/CXCL9, monokine induced by IFN‐γ; MIP‐1α/CCL3, macrophage inflammatory protein; MIP‐1β/CCL4, macrophage inflammatory protein; RANTES/CCL5, regulated on activation normal T‐cell expressed and secreted; *R*_s_, Spearman’s rank‐order correlation coefficient.

Abbreviations: GM‐CSF, granulocyte‐macrophage colony‐stimulating factor; IFN‐γ, interferon gamma; IL‐10, interleukin‐10; IL‐13, interleukin‐13; IL‐15, interleukin‐15; IL‐17, interleukin‐17; IL‐18, interleukin‐18; IL‐2, interleukin‐2; IL‐4, interleukin‐4; IL‐5, interleukin‐5; IL‐6, interleukin‐6; IL‐7, interleukin‐7; LLOQ, lower limit of quantification; TNF‐α, tumor necrosis factor; ULOQ, upper limit of quantification.

^a^×4‐fold dilution.

^b^ *p*‐Value interpreted according to the Holm–Bonferroni corrected significance level.

^c^All correlations were significant at *α* = 0.05.

^d^Significant when *α* = 0.05 but insignificant when the Holm‐Bonferroni correction was applied.

### 3.2. Cytokine Profile in TB Patients and Individuals With LTBI With Positive QuantiFERON‐TB Gold Plus Results

The concentration of 13/20 cytokines (65%) differed significantly between 37 ATB patients and 57 LTBI patients. Twelve cytokines showed significantly higher concentrations in the LTBI group (IFN‐γ, IL‐2, IL‐4, IL‐7, IL‐10, IL‐13, IL‐15, IL‐17, MCP‐1/CCL2, MIP‐1α/CCL3, MIP‐1β/CCL4 and RANTES/CCL5), while only MIG/CXCL9 had significantly higher concentrations in the ATB group (Table 3).

**Table 3:** Comparison of concentration of 20 cytokines between active tuberculosis (ATB) and latent tuberculosis infection (LTBI) patients.

Cytokine TB_max_ (pg/mL)	ATB (*n* = 37)	LTBI (*n* = 57)	Mann–Whitney *U* test *p*‐value^a^
GM‐CSF	18.3, 16.0–21.3 (7.79–40.4)	34.8, 11.2–45.2 (5.74–73.1)	0.037^b^
IFN‐γ	171, 120–217 (36.5–558)	414, 301–587 (120–1359)	<0.001
IL‐2	124, 92.4–148 (33.7–541)	202, 126–359 (34.1–1387)	0.002
IL‐4	20.2, 17.5–22.5 (9.73–30.0)	49.7, 21.7–61.3 (13.4–177)	<0.001
IL‐5	499, 291–565 (151–929)	557, 208–820 (108–1228)	0.604
IL‐6	1870, 495–2383 (66.3–12,155)	1717, 893–2807 (42.0–25,000)	0.268
IL‐7	13.8, 10.8–16.8 (7.74–48.6)	45.6, 36.4–72.8 (9.58–208)	<0.001
IL‐8/CXCL8	56,000, 17,239–56,000 (3952–56,000)	44,175, 26,327–56,000 (4738–56,000)	0.423
IL‐10	12.3, 7.94–19.4 (2.75–101)	24.2, 16.5–32 (9.23–89.4)	<0.001
IL‐13	2.15, 1.43–3.21 (0.683–67.1)	3.51, 1.92–9.35 (1.10–97.6)	0.002
IL‐15	507, 476–538 (314–678)	1229, 597–1434 (355–2899)	<0.001
IL‐17	105, 91.4–110 (43.5–156)	291, 82.7–372 (49.8–760)	0.002
IL‐18	96.2, 65.1–144 (43.9–450)	96.8, 60–120 (20.4–340)	0.286
IP‐10/CXCL10	11,299, 5382–17,528 (1300–140,000)	9568, 4434–23,526 (998–140,000)	0.716
MCP‐1/CCL2	2510, 1614–3534 (296–8104)	4402, 2901–6083 (657–22,106)	<0.001
MIG/CXCL9	15,496, 7882–23,236 (3511–130,000)	6749, 2913–17,200 (1244–111,833)	0.002
MIP‐1α/CCL3	674, 422–1009 (114–1244)	1224, 580–2391 (116–4900)	<0.001
MIP‐1ß/CCL4	1947, 1436–2588 (550–5800)	2648, 2054–3308 (850–5800)	0.002
RANTES/CCL5	6301, 3344–16,685 (391–106,000)	11,796, 8274–23,443 (1846–106,000)	0.003
TNF‐α	1287, 509–2030 (173–6402)	1743, 975–2480 (205–15,263)	0.043^b^

*Note:* Concentrations presented as median, interquartile range (IQR), and range. TB_max_ = the higher concentration of a given cytokine measured in either the TB1 or TB2 antigen tube of the QuantiFERON‐TB Gold Plus test. IL‐8/CXCL8, interleukin‐8; IP‐10/CXCL10, 10 kDa IFN‐γ‐induced protein; MCP‐1/CCL2, monocyte chemoattractant protein; MIG/CXCL9, monokine induced by IFN‐γ; MIP‐1α/CCL3, macrophage inflammatory protein; MIP‐1β/CCL4, macrophage inflammatory protein; RANTES/CCL5, regulated on activation normal T‐cell expressed and secreted.

Abbreviations: ATB, active tuberculosis; GM‐CSF, granulocyte‐macrophage colony‐stimulating factor; IFN‐γ, interferon gamma; IL‐10, interleukin‐10; IL‐13, interleukin‐13; IL‐15, interleukin‐15; IL‐17, interleukin‐17; IL‐18, interleukin‐18; IL‐2, interleukin‐2; IL‐4, interleukin‐4; IL‐5, interleukin‐5; IL‐6, interleukin‐6; IL‐7, interleukin‐7; LTBI, latent tuberculosis infection; TNF‐α, tumour necrosis factor α.

^a^ *p*‐Value interpreted according to the Holm–Bonferroni corrected significance level.

^b^Significant when *α* = 0.05 but insignificant when the Holm–Bonferroni correction is applied.

### 3.3. The Discriminatory Potential of Selected Cytokines in Differentiating ATB and LTBI

IL‐7 had the highest accuracy in distinguishing between ATB and LTBI (AUROC = 95.9%), and at an optimal cut‐off value of 25.7 pg/mL (lower values indicating ATB), it showed balanced Se (94.6%) and Sp (91.2%). Accuracy of IFN‐γ was slightly lower (AUROC = 91.0%), and at an optimal cut‐off value of 250 pg/mL (lower values indicating ATB), it also showed balanced Se (86.5%) and Sp (87.7%). Two other cytokines, IL‐15 and IL‐4, showed good diagnostic accuracy (AUROC 80%–90%), while moderate accuracy (AUROC 70%–80%) was found for IL‐10, MCP‐1/CCL2 and MIP‐1α/CCL3. The remaining analyse cytokines exhibited low discriminatory potential (AUROC <70%; Table 4).

**Table 4:** Discriminatory potential of 13 cytokines whose concentration significantly differed between active tuberculosis (ATB) and latent tuberculosis infection (LTBI) patients.

Cytokine TB_max_ (pg/mL)	AUROC (CI 95%) (%)	Optimal cut‐off value (pg/mL)	Se (CI 95%) (%)	Sp (CI 95%) (%)	LR+ (CI 95%)	LR− (CI 95%)
IL‐7	95.9 (91.5–100)	≤25.7	94.6 (82.3–98.5)	91.2 (81.1–96.2)	10.8 (4.65–25.0)	0.06 (0.02–0.23)
IFN‐γ	91.0 (84.6–97.4)	≤250	86.5 (72.0–94.1)	87.7 (76.8–93.9)	7.04 (3.48–14.3)	0.15 (0.07–0.35)
IL‐15	87.6 (80.7–94.5)	≤616	91.9 (78.7–97.2)	71.9 (59.2–81.9)	3.27 (2.14–5.01)	0.11 (0.04–0.34)
IL‐4	82.6 (74.5–90.8)	≤30.0	100 (90.6–100)	59.6 (46.7–71.4)	2.48 (1.81–3.40)	–∞^a^
IL‐10	76.3 (65.8–86.9)	≤19.4	78.4 (62.8–88.6)	64.9 (51.9–76.0)	2.23 (1.51–3.30)	0.33 (0.18–0.63)
MCP‐1/CCL2	74.8 (64.8–84.7)	≤2579	56.8 (40.9–71.3)	82.5 (70.6–90.2)	3.24 (1.72–6.07)	0.52 (0.36–0.77)
MIP‐1α/CCL3	71.7 (61.6–81.9)	≤1244	100 (90.6–100)	49.1 (36.6–61.7)	1.97 (1.52–2.54)	–∞^a^
IL‐13	69.0 (58.2–79.9)	≤2.2	64.9 (48.8–78.2)	71.9 (59.2–81.9)	2.31 (1.43–3.73)	0.49 (0.31–0.78)
IL‐17	68.9 (57.9–80.0)	≤156	100 (90.6–100)	57.9 (45.0–69.8)	2.38 (1.75–3.22)	–∞^a^
MIG/CXCL9	68.9 (58.3–79.5)	≥14160	67.6 (51.5–80.4)	70.2 (57.3–80.5)	2.27 (1.44–3.58)	0.46 (0.28–0.76)
MIP‐1ß/CCL4	68.9 (57.8–80.1)	≤2121	62.2 (46.1–75.9)	73.7 (61.0–83.4)	2.36 (1.43–3.90)	0.51 (0.33–0.8)
IL‐2	68.7 (57.7–79.6)	≤148	75.7 (59.9–86.6)	70.2 (57.3–80.5)	2.54 (1.64–3.93)	0.35 (0.19–0.63)
RANTES/CCL5	68.0 (55.9–80.1)	≤6504	59.5 (43.5–73.7)	84.2 (72.6–91.5)	3.77 (1.95–7.26)	0.48 (0.32–0.72)

*Note:* TB_max_ = the highest concentration of each cytokine in the TB1 or TB2 antigen tubes of the QuantiFERON‐TB Gold Plus test. MCP‐1/CCL2, monocyte chemoattractant protein; MIG/CXCL9, monokine induced by IFN‐γ; MIP‐1α/CCL3, macrophage inflammatory protein; MIP‐1β/CCL4, macrophage inflammatory protein; RANTES/CCL5, regulated on activation normal T‐cell expressed and secreted; Se, diagnostic sensitivity; Sp, diagnostic specificity.

Abbreviations: AUROC, area under the receiver operating characteristic curve; CI 95%, 95% confidence interval; IFN‐γ, interferon gamma; IL‐10, interleukin‐10; IL‐13, interleukin‐13; IL‐15, interleukin‐15; IL‐17, interleukin‐17; IL‐2, interleukin‐2; IL‐4, interleukin‐4; IL‐7, interleukin‐7; LR−, negative likelihood ratio; LR+, positive likelihood ratio.

^a^–∞ means that the probability quotient of a negative result is very low but cannot be counted because 1 − Se, which is in the denominator of the formula for this quotient, is equal to 0 (Se equal to 100%).

### 3.4. Diagnostic Model Based on IL‐7 and IFN‐γ for Distinguishing ATB From LTBI

Thirteen cytokines whose concentration differed significantly between 37 ATB patients and 57 LTBI patients were offered to the multivariable analysis. The only cytokines that proved to be significantly and independently associated with risk of ATB were IL‐7 (OR = 0.815; CI 95%: 0.735, 0.903; *p*  < 0.001) and IFN‐γ (OR = 0.932; CI 95%: 0.872, 0.996; *p* = 0.039). The logistic model was given by the following formula (*e* stands for Euler’s number, *e* = 2.718):

PATB=e6.9950.20570.007−×IL−×INFγ1+e6.9950.20570.007−×IL−×INFγ.

**Figure 1:**
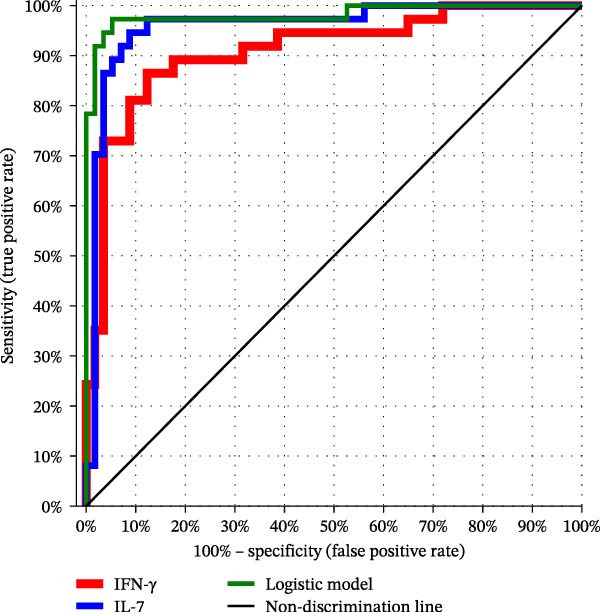
Receiver operating characteristic (ROC) curves of interleukin‐7 (IL‐7), interferon gamma (IFN‐γ), and multiple logistic model based on IL‐7 and IFN‐γ as tests for discrimination between patients with active tuberculosis (ATB; *n* = 37) and latent tuberculosis infection (LTBI; *n* = 57).

The logistic model fit data well (Hosmer–Lemeshow *χ* ^2^ = 14.25, *p* = 0.075; Nagelkerke’s pseudo‐*R* ^2^ coefficient = 0.853) and showed excellent accuracy in distinguishing between ATB and LTBI (AUROC = 98.1%; CI 95%: 95.2%, 99.9%). At the optimal cut‐off value of 0.40 (higher values indicating ATB), Se was 97.3% (CI 95%: 86.2%, 99.5%) and Sp was 94.7% (CI 95%: 85.6%, 98.2%). Also, LR+ (18.5; CI 95%: 6.1, 55.7) and LR− (0.03; CI 95%: 0.01, 0.20) reached satisfactory results. However, the model offered only a little increase in accuracy of classification into ATB and LTBI compared to IL‐7 alone (Figure 1).

### 3.5. Cytokine Profile in ATB Patients With Negative QuantiFERON‐TB Gold Plus Results and Control Group

To investigate to what extent the cytokine profile could be useful in patients with negative QFT‐Plus results, cytokine concentrations of 10 ATB/QFT‐Plus‐negative patients were compared with cytokine concentrations of 51 patients from the control group. The concentrations of 9/20 cytokines (45%) differed significantly. Eight cytokines showed significantly higher concentrations in the control group (IFN‐γ, IL‐4, IL‐7, IL‐10, IL‐15, IL‐17, RANTES/CCL5 and TNFα). Compared to the differences between ATB and LTBI groups, the set of cytokines with significantly different concentrations was smaller but mostly overlapped. Five cytokines (IL‐2, IL‐13, MCP‐1/CCL2, MIP‐1α/CCL3 and MIP‐1β/CCL4) were not significantly different in this analysis, while TNFα was significantly different only in this analysis. MIG/CXCL9 retained its significantly higher concentration in the ATB/QFT‐negative patients (Table 5).

**Table 5:** Comparison of concentrations of 20 cytokines between 10 patients with active tuberculosis and negative QuantiFERON‐TB Gold Plus results (ATB/QFT‐Plus‐negative group) and 51 healthy control patients (control group).

Cytokine TB_max_ (pg/mL)	ATB/QFT‐plus‐negative (*n* = 10)	Control group (*n* = 51)	Mann–Whitney *U* test *p*‐value^a^
GM‐CSF	14.2, 9.74–18.1 (7.79–27.6)	22.0, 12.4–36.7 (5.31–53.6)	0.055
IFN‐γ	111, 72.3–147 (36.5–187)	303, 171–411 (50.9–784)	<0.001
IL‐2	64.0, 48.2–77.5 (33.7–123)	110, 71.7–147 (27.4–262)	0.014^b^
IL‐4	15.6, 12.0–20.9 (9.73–22.6)	53.1, 22.8–89.7 (8.90–177)	<0.001
IL‐5	262, 218–438 (151–568)	317, 176–575 (28.6–1367)	0.661
IL‐6	297, 207–1829 (69.2–2444)	901, 331–2491 (7.37–9003)	0.112
IL‐7	11.6, 10.8–13.8 (7.74–25.7)	72.8, 41.5–118 (10.9–172)	<0.001
IL‐8/CXCL8	15,702, 7048–56,000 (5524–56,000)	24,446, 9960–52,606 (2460–56,000)	0.806
IL‐10	8.27, 7.94–13.9 (4.67–43.6)	18.5, 12.7–34.3 (5.46–78.8)	0.003
IL‐13	1.24, 0.683–1.79 (0.683–2.15)	1.52, 1.10–2.32 (0.089–7.50)	0.279
IL‐15	415, 360–498 (314–515)	1115, 597–1721 (452–2774)	<0.001
IL‐17	82.2, 55.1–107 (43.5–110)	253, 103–338 (38.0–660)	0.001
IL‐18	78.9, 61.1–100 (45.4–203)	74.1, 47.3–105 (16.3–217)	0.442
IP‐10/CXCL10	4317, 2293–6589 (1300–12,821)	2477, 1216–6760 (394–46,334)	0.263
MCP‐1/CCL2	1565, 734–1799 (370–3534)	2758, 888–4374 (74–10,780)	0.189
MIG/CXCL9	9586, 6452–14,160 (4545–17,898)	2453, 1218–4870 (412–17,180)	<0.001
MIP‐1α/CCL3	339, 219–667 (114–1177)	440, 241–1165 (9.53–4900)	0.365
MIP‐1ß/CCL4	731, 686–1444 (550–2588)	1417, 989–2532 (521–5800)	0.017^b^
RANTES	5444, 1423–6362 (1011–22,264)	10,430, 6354–16,673 (2099–106,000)	0.002
TNF‐α	496, 466–1297 (173–3511)	1684, 864–2999 (300–7455)	0.003

*Note:* Concentrations presented as median, interquartile range (IQR), and range. ATB QFT‐Plus (−) = tuberculosis with negative QuantiFERON‐TB Gold Plus result; non‐TB/LTBI QFT‐Plus (−) = non tuberculosis and latent tuberculosis infection with negative QuantiFERON‐TB Gold Plus result; TB_max_ = the highest [pg/mL] value of each cytokine among the 20 tested in the TB1 or TB2 antigen tubes of the QuantiFERON‐TB Gold Plus test. IL‐8/CXCL8, interleukin‐8; IP‐10/CXCL10, 10 kDa IFN‐γ‐induced protein; MCP‐1/CCL2, monocyte chemoattractant protein; MIG/CXCL9, monokine induced by IFN‐γ; MIP‐1α/CCL3, macrophage inflammatory protein; MIP‐1β/CCL4, macrophage inflammatory protein; RANTES/CCL5, regulated on activation normal T‐cell expressed and secreted.

Abbreviations: GM‐CSF, granulocyte‐macrophage colony‐stimulating factor; IFN‐γ, interferon gamma; IL‐10, interleukin‐10; IL‐13, interleukin‐13; IL‐15, interleukin‐15; IL‐17, interleukin‐17; IL‐18, interleukin‐18; IL‐2, interleukin‐2; IL‐4, interleukin‐4; IL‐5, interleukin‐5; IL‐6, interleukin‐6; IL‐7, interleukin‐7; TNF‐α, tumour necrosis factor.

^a^ *p*‐Value interpreted according to the Holm–Bonferroni corrected significance level.

^b^Significant when *α* = 0.05 but insignificant when the Holm–Bonferroni correction is applied.

**Figure 2:**
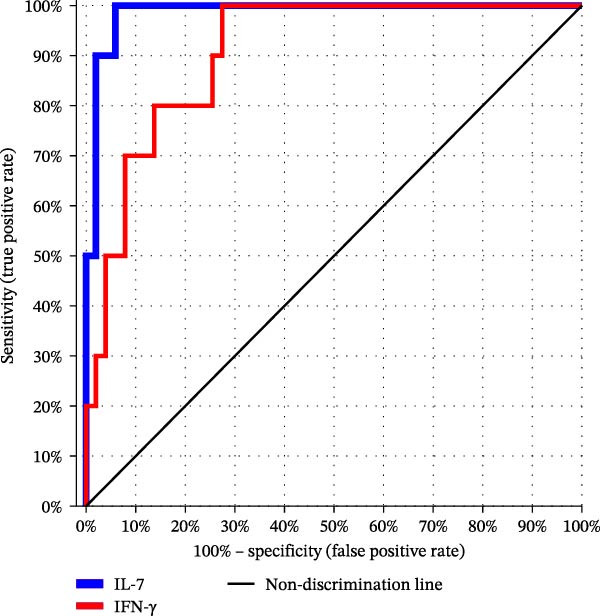
Receiver operating characteristic (ROC) curves of interleukin‐7 (IL‐7) and interferon gamma (IFN‐γ) as tests for discrimination between patients with active tuberculosis (ATB) and negative QuantiFERON‐TB Gold Plus results (*n* = 10) and healthy control patients (*n* = 51).

Analogically to the previous analysis, the aforementioned 9 cytokines were offered to multivariable logistic regression. Only IL‐7 turned out to be significantly associated with the risk of ATB in the multivariable analysis (OR = 0.777, CI 95%: 0.648, 0.931; *p* = 0.006). Moreover, it proved to have excellent accuracy in discriminating between ATB/QFT‐Plus‐negative and healthy patients (AUROC = 98.6%; CI 95%: 96.2%, 99.9%), and at the optimal cut‐off value of 25.7 pg/mL (lower values indicating ATB), it showed Se of 100% (CI 95%: 72.2%, 100%) and Sp of 94.1% (CI 95%: 84.1%, 98.0%). These figures were considerably better than for IFN‐γ (AUROC = 90.8%; CI 95%: 82.8%, 98.7%; at an optimal cut‐off value of 187 pg/mL: Se = 100%; CI 95%: 72.2%, 100%, and S*p*  = 72.5%; CI 95%: 59.1%, 82.9%; Figure 2).

## 4. Discussion

The diagnosis of LTBI and its differentiation from ATB remain a significant challenge in TB immunodiagnostics [16]. Current assays only identify exposure to *M. tuberculosis*, while their ability to unequivocally distinguish LTBI from ATB remains limited [17]. Therefore, there is a need to look for new biomarkers to differentiate the two conditions more accurately and to assess the risk of infection progression.

The cytokine profiles obtained in the present exploratory study confirm that the immune response in LTBI and ATB patients differs both qualitatively and quantitatively. Elevated levels of IFN‐γ and cytokines associated with T‐cell activation and proliferation (IL‐2, IL‐7 and IL‐15) were observed in LTBI patients, which may reflect a controlled immune response favouring the maintenance of infection in the latent phase. The concomitant presence of regulatory cytokines (IL‐10 and IL‐13) and pro‐inflammatory cytokines (IL‐17) indicates the balanced nature of the immune response, which may prevent the progression of infection to ATB [16, 18–23]. In turn, higher concentrations of MIG/CXCL9 in ATB patients suggest its potential role in identifying active disease and distinguishing it from latent infection [24, 25].

Among the cytokines analysed, IL‐7 showed a particularly high diagnostic value. This cytokine, produced mainly by marrow and thymus lining cells, as well as keratinocytes, dendritic cells, neurones and endothelial cells, and not by T lymphocytes, plays a key role in the proliferation, differentiation, and maintenance of T‐lymphocyte homeostasis [19, 20, 26, 27].

In the present study, IL‐7 shoved very high accuracy in distinguishing between ATB and LTBI patients as well as between ATB/QFT‐negative patients and healthy controls, outperforming IFN‐γ, which is the basis of commercial IGRA tests [16, 23]. The increase in IL‐7 may reflect the more stable and long‐lasting immune response mechanisms characteristic of the latent state, in contrast to IFN‐γ, whose expression is strictly dependent on the activation of effector T‐cells. In particular, IL‐7 showed strong individual diagnostic performance, while a combined logistic regression model incorporating IL‐7 and IFN‐γ achieved very high internal accuracy in distinguishing between the studied groups.

The number of antigen‐specific memory T‐cells may be reduced in ATB patients, suggesting a disruption of T‐cell homeostasis dependent on the IL‐7/IL‐7R pathway [26, 27]. Differences in the expression of IL‐7 indicate their potential as biomarkers of both LTBI and ATB [17].

Despite these encouraging findings, several important limitations must be considered when interpreting the results. The study has some limitations, including the relatively small group size and the analysis of cytokines only in plasma stimulated with ESAT‐6 and CFP‐10 antigens. In addition, the lack of a gold standard for the diagnosis of LTBI further complicates the interpretation of the results [4].

Importantly, the performance estimates reported in this study are based on a single‐centre cohort and represent internal, non‐externally validated results. These findings should therefore be interpreted as indicating a strong cohort‐specific signal rather than definitive diagnostic accuracy.

As such, they may reflect cohort‐specific characteristics, including patient selection, disease spectrum and modelling within the same dataset. Despite the application of robust statistical methods, including correction for multiple comparisons and multivariable logistic regression, the possibility of model optimism and overfitting cannot be excluded, particularly given the relatively limited sample size and the high dimensionality of the cytokine panel.

Consequently, the very high diagnostic accuracy observed for IL‐7 and for the combined IL‐7/IFN‐γ model should not be interpreted as evidence of immediate clinical applicability. Rather, these results should be considered hypothesis‐generating and indicative of a potentially relevant biological signal that warrants further investigation.

External validation in independent and geographically diverse cohorts is therefore essential before any clinical implementation can be considered. Only such studies can determine whether the observed diagnostic performance is robust, reproducible and generalisable across different populations and epidemiological settings.

Nevertheless, our data suggest that IL‐7 may represent a promising biomarker for differentiating LTBI and ATB. Its integration with IFN‐γ in IGRA–based approaches may contribute to the development of a new generation of immunodiagnostic tools aimed at a more precise assessment of infection status and risk of disease progression.

Despite these limitations, the results support a biologically plausible role of IL‐7 in T‐cell homeostasis and immune regulation in TB infection and highlight its potential relevance within multi‐biomarker diagnostic strategies rather than as a standalone assay.

## 5. Conclusion

In conclusion, the findings of this exploratory study indicate that IL‐7 may represent a biologically relevant candidate biomarker for differentiating LTBI and ATB, particularly when combined with IFN‐γ. Given the exploratory nature of the study, further multicentre studies with external validation are required to confirm these observations and to assess the potential clinical utility of IL‐7, including its application in multi‐biomarker strategies.

## Funding

This research was supported by the statutory funds of the Institute of Tuberculosis and Lung Diseases under Statutory Activity 2023–2025 (Research Topic 1, Task 1.8).

## Conflicts of Interest

The authors declare no conflicts of interest.

## Data Availability

The data that support the findings of this study are available from the corresponding author upon reasonable request.
